# Pitfall in the Diagnosis of Diabetes Insipidus and Pregnancy

**DOI:** 10.1155/2017/7879038

**Published:** 2017-07-27

**Authors:** Melissa Sum, Jessica B. Fleischer, Alexander G. Khandji, Sharon L. Wardlaw

**Affiliations:** ^1^Department of Medicine, College of Physicians and Surgeons, Columbia University, New York, NY, USA; ^2^Department of Radiology, College of Physicians and Surgeons, Columbia University, New York, NY, USA

## Abstract

Diabetes insipidus (DI) during pregnancy and the perinatal period is an uncommon medical problem characterized by polyuria and excessive thirst. Diagnosis of DI may be overlooked in the setting of pregnancy, a time when increased water intake and urine output are commonly reported. We report two cases: one of transient DI in a young woman during her third trimester of twin pregnancy in association with acute fatty liver and hypertension and one of postpartum DI secondary to Sheehan syndrome from rupture of a splenic artery aneurysm. These cases illustrate the spectrum with which DI related to pregnancy and delivery can present and highlight the difficulty in making the diagnosis since the symptoms are often initially overlooked.

## 1. Introduction

Diabetes insipidus (DI) during pregnancy is an uncommon medical problem estimated to occur in two to six of 100,000 pregnancies [1]. One possible explanation is release of vasopressinase, a cysteine aminopeptidase, from the placenta leading to a fourfold increase in the rate of breakdown of arginine vasopressin (AVP) [2]. AVP regulates water reabsorption in the kidney and a decreased level leads to water loss. Other cases have been caused by uncommon hypothalamic-pituitary disorders leading to deficient secretion of AVP [3–5]. Timely diagnosis can be challenging because symptoms of polyuria, defined as a urine output exceeding 3 liters per day, and polydipsia may be attributed to the state of pregnancy. We present a case of transient DI in a woman during her third trimester of twin pregnancy and a second case of postpartum DI secondary to Sheehan syndrome to illustrate two different causes of DI associated with pregnancy and to highlight the difficulty in making a diagnosis of DI in the peri- and postpartum states.

## 2. Case  1

A 28-year-old para 1 woman was admitted in the 33rd week of gestation for hypertension and elevated liver enzymes. Concern for acute fatty liver of pregnancy or early preeclampsia prompted Cesarean section, which yielded two viable female infants. Her prenatal course was uncomplicated until week 25, when she developed polyuria and polydipsia leading to intake of 12 liters of water daily. The quantity and significance of the polyuria were initially not recognized and attributed to the gestational state.

**Figure 1:**
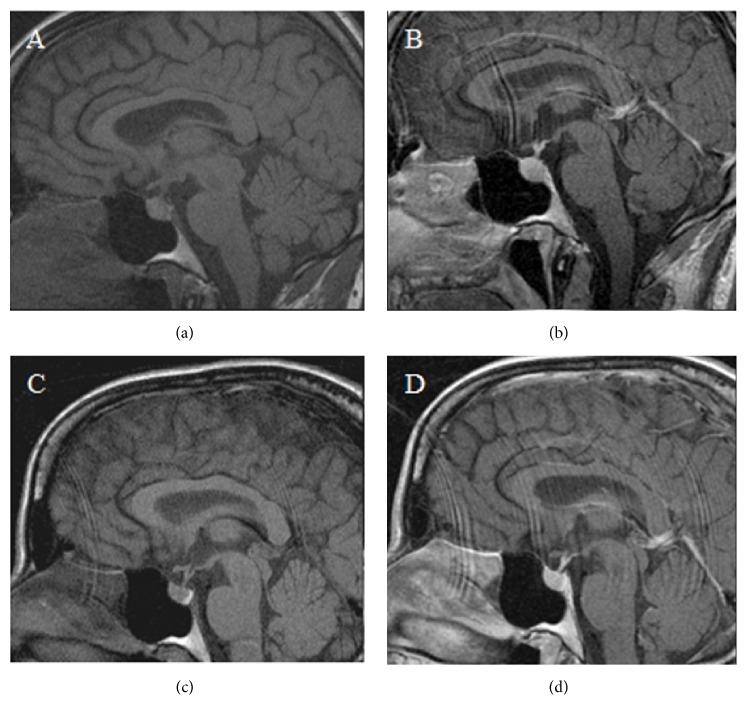
(a, b) Pre- and postcontrast brain MRI images of case 1 show that posterior pituitary bright spot is not visualized. (c, d) Pre- and postcontrast brain MRI images of case 1 at four months postpartum show return of posterior pituitary bright spot.

On examination, her temperature was 37.1°C, blood pressure was 128/76 mmHg, and pulse was 90 beats per minute with normal skin turgor and no peripheral edema. Admission laboratory data included elevated liver enzymes and normal serum sodium that increased to 154 mmol/L after C-section (Table 1). Postpartum she had polyuria up to 1000 mL/h, 24-hour fluid intake was 9.5 liters, and urine output was 10.6 liters. She exhibited severe thirst, ongoing dilute polyuria, and elevated serum osmolality during her water deprivation test unresponsive to 8-arginine vasopressin (Pitressin) but responsive to 1-deamino-8-D-arginine-vasopressin (DDAVP), which provided the patient with substantial relief (Table 2). Liver function tests improved at five days postpartum, but DI persisted, requiring DDAVP 10 *μ*g intranasally twice a day. Brain MRI (Figures 1(a) and 1(b)) showed loss of the normal hyperintense posterior pituitary signal consistent with AVP depletion. By eighteen days postpartum, her polyuria and polydipsia resolved, and DDAVP was discontinued. Urine osmolality was 687 mOsm/kg. Four months postpartum, repeat MRI (Figures 1(c) and 1(d)) showed return of the hyperintense posterior pituitary signal, consistent with AVP repletion.

## 3. Case  2

A 35-year-old nulliparous woman in her 29th week of an uncomplicated gestation presented for severe, generalized abdominal pain. Ultrasound was concerning for pelvic free fluid. She subsequently decompensated and was rushed to the emergency room for resuscitation. She required fluid boluses, pressors, intubation, and emergent C-section. Hemorrhage ensued, and she underwent an exploratory laparotomy where her ruptured splenic artery aneurysm was ligated and spleen was removed, resulting in hemostasis. She required 18 units of transfused packed red blood cells. The baby did not survive.

**Figure 2:**
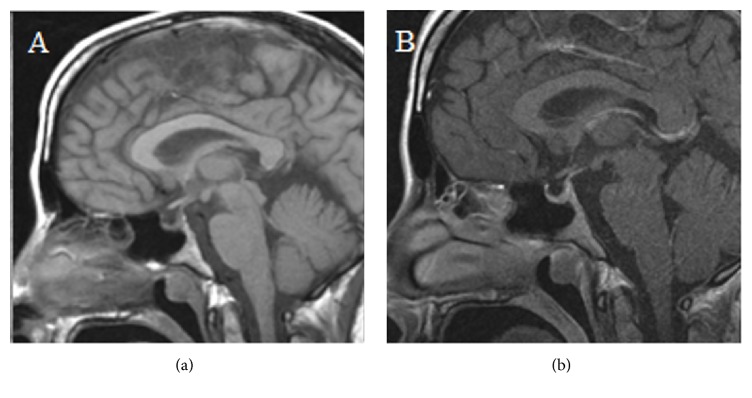
(a, b) Pre- and postcontrast brain MRI images of case 2 show that posterior pituitary bright spot is not visualized and that the anterior pituitary appears small for age and postpartum state.

Her past medical history was notable for polycystic kidney disease diagnosed in adulthood and a family history of polycystic kidney disease in her mother and brother. Postoperatively, her exam was notable for a soft abdomen and closed midline incision. Ten days afterwards while still in-house, she noted onset of polyuria and polydipsia initially attributed to fluid shifts from her resuscitation, postpartum, and postoperative state. However, those symptoms worsened upon discharge. She reported strong desire for cold fluids and polyuria that interrupted sleep. Four weeks later, she was admitted for wound infection and noted to have up to 450 mL/hr of urine output with osmolality 101 mOsm/kg and elevated serum osmolality 297 mOsm/kg. She responded well to DDAVP 10 *μ*g  intranasally with concentration of her urine and relief of symptoms. She was subsequently maintained on nightly intranasal DDAVP. She denied headache or vision problems. Results of her pituitary hormone panel included TSH 4.05 U/mL, free T4 0.9 ng/dL, PRL 25 ng/mL, FSH 1.1 [<15.0 mIU/mL], LH 0.5 [<15.0 mIU/mL], estradiol 121 [100–400 pg/mL], and cortisol 7.9 *μ*g/dL. She had no symptoms suggesting hypothyroidism or adrenal insufficiency. Brain MRI (Figures 2(a) and 2(b)) showed lack of normal hyperintense posterior pituitary signal and small pituitary size for age and postpartum state. Repeat morning cortisol was 17.3 *μ*g/dL. She had eventual resumption of menses. Two years later, she had a spontaneous pregnancy and uneventful delivery of a full-term healthy baby boy. Her DI has persisted and remains controlled on DDAVP.

## 4. Discussion

Diabetes insipidus is characterized by polyuria and polydipsia. Since these symptoms are nonspecific and may be attributed to the gestational state, DI during pregnancy is often overlooked and diagnosis is delayed. Indeed, in normotensive healthy human pregnant subjects, the osmotic threshold for AVP release and thirst perception is decreased compared to nonpregnant subjects [6]. The change in osmotic threshold may be mediated by human chorionic gonadotropin (hCG), as administration of hCG to women during the menstrual luteal phase has been shown to induce similar threshold changes for ADH release and thirst [7, 8]. As a result of these set point changes, plasma osmolality in normal pregnancy decreases to about 270 mosmol/kg and plasma sodium concentration decreases 4 to 5 meq/L below nonpregnancy levels [9]. In addition to a physiologic decrease in threshold for thirst perception, urinary frequency, defined as voiding more than 7 times per day, and nocturia, defined as voiding more than or equal to 2 times per night, are common and can affect 80–95% of pregnant women [10–12]. Yet in DI, the polyuria is generally of rapid onset and defined by abnormally high volumes of dilute urine exceeding three liters per day and the thirst can be intense.

In general, the evaluation of patients with suspected DI begins with a detailed history including rate of onset of polyuria, appearance of urine, and measurement of fluid intake and urine output. Typical findings include increased serum osmolality though, notably, the osmolality may be comparable to that of a nonpregnant woman in light of the decreased physiologic set point that occurs in pregnancy, elevated serum sodium concentration, and decreased urine osmolality when fluid is restricted. An increase in urine osmolality of at least 50% in response to DDAVP is consistent with central versus nephrogenic DI. The water restriction test must be performed in a monitored setting because potentially severe volume depletion and hypernatremia can occur in patients with significant polyuria. During pregnancy, the test is generally not recommended or must be undertaken with significant caution and low threshold to terminate since dehydration can lead to uteroplacental insufficiency.

DI associated with pregnancy can result from decreased AVP production associated with pathological processes involving the hypothalamus and pituitary such as lymphocytic hypophysitis and infundibulitis or postpartum hemorrhage or from increased AVP destruction secondary to increased vasopressinase activity by means of enhanced placental production or decreased clearance in the setting of liver dysfunction. Patients with prepregnancy mild subclinical central DI that may have been present due to prior hypothalamic/pituitary disease experience worsening of symptoms which are unmasked by the increased vasopressinase activity during gestation. Furthermore, the marked increase in glomerular filtration rate during pregnancy may worsen preexisting subclinical nephrogenic DI.

Transient DI of pregnancy attributed to increased vasopressinase activity typically presents in the third trimester. Loss of the hyperintense posterior pituitary signal indicating a decrease in AVP reserves has been reported [13]. Our first case also documents return of the hyperintense posterior pituitary signal following resolution of gestational DI. There appears to be an association with hepatic abnormalities perhaps because liver dysfunction results in decreased hepatic degradation of vasopressinase [14]. Prior gestational history may also be notable for occurrence of polyuria and polydipsia [13]. In our first patient, twin pregnancy with an extra placenta likely contributed to increased vasopressinase production while hepatic abnormalities hindered its clearance, leading to DI. Her diagnosis was delayed until the postpartum setting when her urine output was quantified. The water deprivation test might not have been needed since the patient was already hypernatremic and a vasopressin challenge could have been done. But notably, during the deprivation test, our patient had better response to DDAVP than AVP, consistent with involvement of vasopressinase in the pathogenesis of her DI, as vasopressinase cleaves the N-terminus of AVP and oxytocin and DDAVP lacks an amino group, thereby protecting it from degradation.

Our second case is the first report of isolated DI secondary to Sheehan syndrome from rupture of a splenic artery aneurysm. Sheehan syndrome is a well-known complication of postpartum hemorrhage that typically manifests with anterior pituitary hormone deficiencies including lactation failure and amenorrhea. Rare cases of DI in the setting of Sheehan syndrome have been reported, nearly all of which involved anterior pituitary hypofunction; we found only one case with isolated DI [3]. Only one reported case of Sheehan syndrome was secondary to ruptured splenic artery aneurysm [15]. Our patient's history of polycystic kidney disease may have predisposed her to aneurysm development [16]. Despite a known history of large volume blood loss placing her at risk of Sheehan syndrome, her diagnosis of DI was delayed until her readmission for a wound infection when the amount of polyuria was recorded.

These cases illustrate the spectrum with which DI related to pregnancy and delivery can present and highlight the difficulty of making the diagnosis. This report does not illustrate the full range of etiologies of DI and pregnancy reported in literature but does highlight 2 important causes including one case of decreased AVP in the setting of likely increased vasopressinase release and decreased hepatic degradation and a second case of deficient AVP secretion. The patient courses described here are also consistent with other reports of delayed diagnoses, as the diagnosis of DI is often not considered since urinary frequency is common during gestation. In both cases reported here, quantification of urine output was helpful in alerting physicians about the presence of true polyuria. The treatment of choice for DI is DDAVP. Recognition and treatment of DI are important to prevent dehydration, hypernatremia, and oligohydramnios and to alleviate the distress associated with unrelenting polyuria and polydipsia. Furthermore, for pregnant patients on DDAVP receiving parenteral fluids in the peripartum period, recognition of the risk of hyponatremia and avoidance of hypotonic fluids are important. As such, the diagnosis and treatment of DI during pregnancy merit special consideration.

## Figures and Tables

**Table 1:** Laboratory data for Case 1.

Laboratory data on admission		Laboratory data, 1 day postpartum	
Sodium [135–145 mmol/L]	137	Sodium	147
BUN [7–20 mg/dL]	17	Serum osmolality [275–295 mOsm/kg]	328
Creatinine [0.5–0.9 mg/dL]	1.0	Urine osmolality [500–800 mOsm/kg]	116
AST [7–41 U/L]	1337	TSH [0.34–4.25 U/mL]	0.79
ALT [12–38 U/L]	1359	Free T4 [0.8–1.8 ng/dL]	1.1
Total bilirubin [0.30–1.3 mg/dL]	3.4	Prolactin [1–25 ng/mL]	135
Alkaline phosphatase [33–96 U/L]	496	Cortisol [6.2–19.4 *µ*g/dL]	12.1

**Table 2:** Pitressin and DDAVP challenge for Case 1.

	Time	Urine output	Urine specific gravity	Urine osm [mOsm/kg]	Serum osm [mOsm/kg]	Sodium [mmol/L]

5 units of SQ Pitressin	1400 h	500 mL		116	328	
	1600 h	600 mL		87	301	
	1700 h	600 mL	1.005			141
	1800 h	150 mL	1.005		301	144
	1900 h	350 mL				

10 *µ*g of intranasal DDAVP	2100 h	700 mL				
	2200 h					135
	0000 h					
	0200 h	75 mL				
	0400 h	60 mL				
	0600 h	60 mL	1.020			131
